# Phosphorylation Regulation of a Histone-like HU Protein from *Deinococcus radiodurans*

**DOI:** 10.2174/0929866529666220819121911

**Published:** 2022-11-08

**Authors:** Jinfeng Hou, Jingli Dai, Zijing Chen, Yudong Wang, Jiajia Cao, Jing Hu, Shumai Ye, Yuejin Hua, Ye Zhao

**Affiliations:** 1 MOE Key Laboratory of Biosystems Homeostasis & Protection, College of Life Sciences, Zhejiang University, Hangzhou 310000, China

**Keywords:** Nucleoid-association protein, histone-like protein, deinococcus, phosphorylation, DNA binding, DrHU

## Abstract

***Background*:** Histone-like proteins are small molecular weight DNA-binding proteins that are widely distributed in prokaryotes. These proteins have multiple functions in cellular structures and processes, including the morphological stability of the nucleoid, DNA compactness, DNA replication, and DNA repair. *Deinococcus radiodurans*, an extremophilic microorganism, has extraordinary DNA repair capability and encodes an essential histone-like protein, DrHU.

***Objective*:** We aim to investigate the phosphorylation regulation role of a histone-like HU protein from *Deinococcus radiodurans.*

***Methods*:** LC-MS/MS analysis was used to determine the phosphorylation site of endogenous DrHU. The predicted structure of DrHU-DNA was obtained from homology modeling (Swiss-model) using *Staphylococcus aureus* HU-DNA structure (PDB ID: 4QJU) as the starting model. Two types of mutant proteins T37E and T37A were generated to explore their DNA binding affinity. Complemented-knockout strategy was used to generate the *ΔDrHU/pk-T37A* and *ΔDrHU/pk-T37E* strains for growth curves and phenotypical analyses.

***Results and Discussion*:** The phosphorylation site Thr37, which is present in most bacterial HU proteins, is located at the putative protein-DNA interaction interface of DrHU. Compared to the wild-type protein, one in which this threonine is replaced by glutamate to mimic a permanent state of phosphorylation (T37E) showed enhanced double-stranded DNA binding but a weakened protective effect against hydroxyl radical cleavage. Complementation of T37E in a DrHU-knockout strain caused growth defects and sensitized the cells to UV radiation and oxidative stress.

***Conclusions*:** Phosphorylation modulates the DNA-binding capabilities of the histone-like HU protein from *D. radiodurans*, which contributes to the environmental adaptation of this organism.

## 1 INTRODUCTION

A number of small molecular-weight proteins commonly referred to as histone-like proteins involved in genome integrity have been identified in eubacteria. As the name suggests, these proteins are nucleoid-association proteins because of their functional similarity to eukaryotic histone proteins. Based on their abundance in living cells and capacity to bind DNA, histone-like proteins are associated with nucleoid stability, DNA compaction, DNA replication, and DNA repair [1-3].

HU is a histone-like protein that is essential for some gram-positive bacteria. In *Streptococcus pneumoniae*, HU is required for cell viability, and conditional inactivation of this protein impairs DNA supercoiling *in vivo* [4]. In contrast, *Escherichia coli* cells only show modest phenotypes in the absence of HU, which might be due to the expression of redundant histone-like proteins [5]. In addition, HU proteins have been proposed to play important roles in the regulation of multiple cellular processes, such as cell motility, the SOS response, cell division, and virulence gene expression [6-10]. In the case of *E. coli*, HU is involved in the regulation of SOS genes and is able to displace the LexA repressor from the SOS box [11].

**Figure 1:**
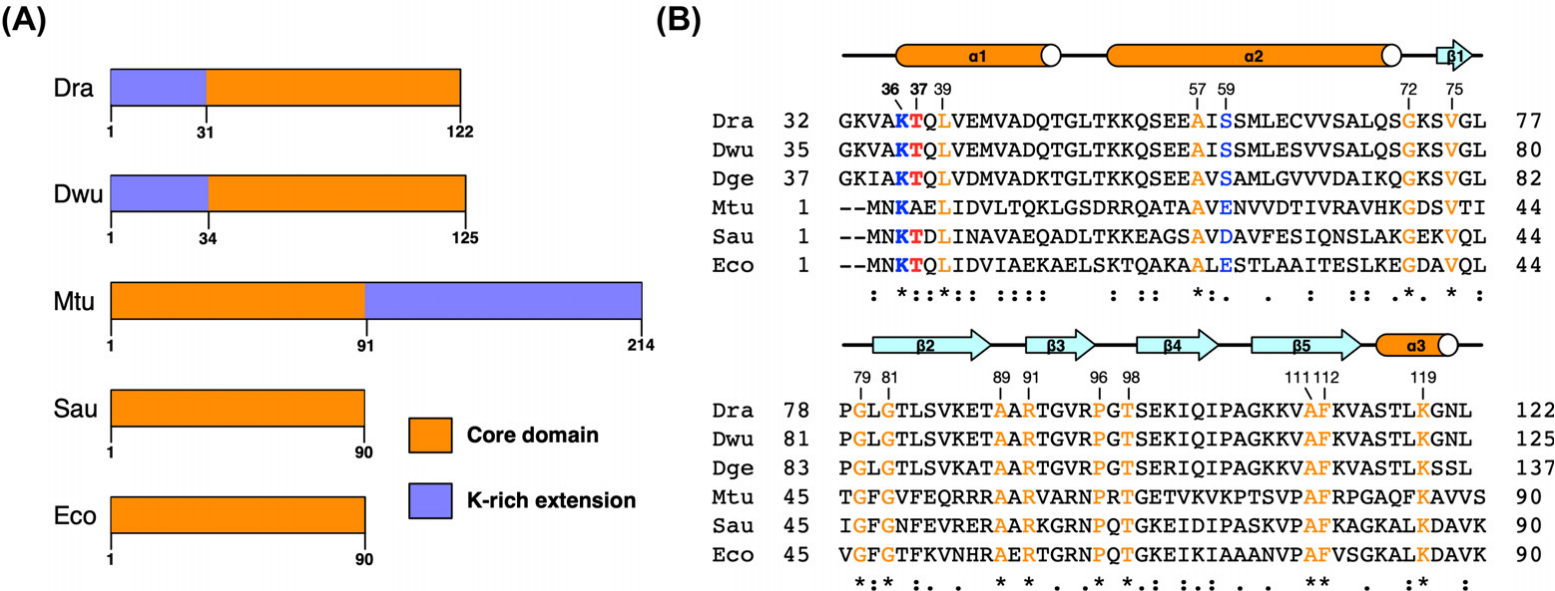
Alignment of HU proteins from different organisms. (**A**) Schematic presentation of HU proteins. The core domain and lysine-rich N- or C-terminal extensions are shown in orange and blue, respectively. (**B**) Structure-based sequence alignment of the core domain of HU proteins. HU proteins from *Deinococcus* species, including *Deinococcus radiodurans* and *Deinococcus wulumuqiensis*, and *Deinococcus geothermalis* are denoted by Dra, Dwu and Dge, respectively. HU proteins from *Mycobacterium tuberculosis*, *Staphylococcus aureus*, and *Escherichia coli* are denoted by Mtu, Sau and Eco, respectively. The phosphorylation site is highlighted in red. Two residues forming salt bridge interactions are colored blue. Conserved residues are highlighted in orange.

As a DNA-binding protein, HU can bind to different types of DNA in a non-specific manner. However, for canonical HU proteins, binding affinity for abnormal DNA, such as Holliday junctions, DNA forks, and gapped or nicked duplex DNA, appears to be higher than that for regular duplex DNA [12]. These DNA structures represent intermediates in DNA recombination and repair. Increasing evidence shows that HU protein binding protects such DNA structures from hydroxyl radical cleavage or nuclease degradation [13]. HU from *Mycobacterium smegmatis* promotes the DNA end-joining reaction, an important DNA double-strand break repair pathway, which is consistent with the proposed DNA repair function of HU proteins [14]. Sequence alignment has revealed that HU proteins contain a relatively conserved core of approximately 90 amino acids and can have extensions at their N- or C-terminus (Figure **1A**). Most of these extensions are rich in lysine residues and participate in the modulation of HU DNA binding [15, 16]. Thus far, several structures of apo HU and HU-DNA complexes for various bacteria have been solved by X-ray crystallography and NMR [17-20]. Consistent with the in-solution dimeric state, HU forms a V-shaped core structure containing an intertwined α-helical body and two flexible protruding β-arms, which undergo conformational changes upon DNA minor-groove binding. Moreover, several conserved arginine residues important for DNA binding and HU-protein interactions have been identified [21, 22]. For example, the β-arm residue Arg55 is essential for the DNA binding of HU protein from *Bacillus stearothermophilus* [23].

*Deinococcus radiodurans*, a non-spore-forming gram-positive bacterium, is well known for its extreme resistance to environmental stresses, including ionizing radiation, oxidative stress, and DNA damage. It has been shown that the robustness of this bacterium is the result of multiple mechanisms, *e.g*., enhanced DNA repair capabilities, novel DNA damage responses, effective antioxidation systems, and cellular protection of damaged DNA and proteins [24-26]. Whole-genome sequence data reveal that *D. radioduran*s encodes one HU protein, DrHU (*DR_A0065*), which is highly expressed from a leaderless mRNA [27]. In contrast to the subtle phenotypes of the *E. coli* HU-knockout strain, DrHU is required for cell viability under normal growth conditions. Progressive depletion of DrHU using a thermosensitive vector causes diffuse and enlarged nucleoid morphology, further leading to cell death [28]. Sequence alignment and biochemical studies indicate that DrHU contains an additional N-terminal 30 amino acids outside the core structure. This N-terminal extension is rich in positively charged lysine residues akin to the C-terminus of histone eukaryotic H1, which significantly modulates DNA-binding specificity [29]. Moreover, prebent DNA and Holliday junctions appear to be preferred by DrHU, with a low affinity for normal duplex DNA [30]. Furthermore, a recent study involving atomic force microscopy demonstrated that both the DNA configuration and protein concentration control the DNA binding and compaction of DrHU. Higher-ordered DrHU-DNA architectures are observed with multimerization at high DrHU concentrations [31].

Posttranslational modifications (PTMs) play fundamental roles in various cellular processes and are critical for appropriate protein functions and biochemical activities. The role of methylation and phosphorylation in histone regulation is well documented [32]. In *D. radioduran*s, lysine succinylation regulates a variety of biological processes and enzymatic activities of important DNA repair proteins, such as PprI and DdrB [33]. Phosphorylation, a common PTM in cells, participates in the activation of the RecA protein, the central recombinase involved in DNA repair in *D. radioduran*s [34]. Despite a limited number of studies, PTM is known to regulate HU proteins in terms of DNA-binding capacity and bacterial pathogenicity [16]. Lysine acetylation and serine/threonine phosphorylation of the *Mycobacterium tuberculosis* HU protein modulate its ability to compact DNA, but mutations of PTM sites reduce the drug-tolerant population, linking PTM of bacterial HU to antibiotic treatment [22, 35]. To investigate the potential mechanism of PTM regulation, we sought to determine the phosphorylation site of DrHU and identified Thr37 adjacent to an almost universally conserved lysine residue. Mimicking HU phosphorylation leads to enhanced duplex DNA binding but reduces cell growth and environmental stress resistance, suggesting the important role of phosphorylation regulation of HU protein in *D. radioduran*s.

## 2 MATERIALS AND METHODS

### 2.1 Strain Construction and Cloning

All strains and plasmids used in this study are listed in Table **S1**. All primers used are given in Table **S2**. The *D. radioduran*s wild-type R1 strain (ATCC13939) and mutants were grown in TGY broth (0.5% tryptone, 0.3% yeast extract, and 0.1% glucose) or on TGY agar plates (1.5% agar) at 30°C. Full-length *DR_A0065* (encoding 122 amino acids) was cloned into the shuttle vector pRADK containing the *D. radioduran*s groEL promoter for complementation. Site-directed mutagenesis was carried out using PrimeSTAR HS DNA polymerase (Takara) to generate complementary plasmids containing T37A and T37E DrHU. The complementation-knockout *ΔDrHU/pk-T37A* and *ΔDrHU/ pk-T37E* strains were constructed by firstly transforming the complementary plasmid into the wild-type R1 strain, followed by the tripartite ligation method, and the genomic *DR_A0065* gene was substituted with the streptomycin gene, as described previously [36]. Briefly, the DNA fragments upstream and downstream of *DR_A0065* were firstly amplified by PCR. Following digestion with BamHI and HindIII, the fragments were ligated to a streptomycin gene, which was predigested with BamHI and HindIII. The triplet ligation product was amplified and transformed into competent cells. The mutants were selected with streptomycin and chloramphenicol, and confirmed by sequencing.

### 2.2 Phosphorylation Site Identification

Identification of the DrHU phosphorylation site was performed using a similar method as described but with modification [33]. Briefly, cells (OD_600_ = 1.0) were harvested by centrifugation at 8000 × g and resuspended in sterile phosphate-buffered saline (PBS) three times. Four volumes of lysis buffer (100 mM NH_4_HCO_3_ (pH 8), 6 M urea, and 0.2% SDS) were added, followed by sonication on ice. After concentration at 20000 × g at 4°C, the protein extracts were subjected to disulfide reduction with 2 mM DTT (56°C, 1 h) and subsequently alkylated with 20 mM iodoacetamide (25°C, 1 h in the dark). Four volumes of ice-cold acetone were incubated with samples at -20°C for 2 h. After centrifugation, the precipitate was collected and washed twice with cold acetone, and the pellet was dried and redissolved in a dissolution buffer containing 0.1 M triethylammonium bicarbonate (TEAB, pH 8.5) and 6 M urea. The supernatant was digested with Trypsin Gold (Promega) at a 1:50 enzyme-to-substrate ratio at 37°C for 16 h, and then the peptides were desalted with a C18 cartridge to remove high levels of urea and dried by vacuum centrifugation. The dried fractions were redissolved in 250 mM acetic acid with 30% acetonitrile, and 1 M HCl was used to adjust the pH to 2.5-3.0. Enrichment was carried out using Phos-select iron affinity gel (Sigma, P9740) following the manufacturer’s instructions. The bound peptides were eluted, dried, and desalted using peptide desalting spin columns (Thermo Fisher, 89852). The enriched phosphopeptide mixture was analyzed using an EASY-nLCTM 1200 UHPLC system (Thermo Fisher) coupled with an Orbitrap Q Extractive HF-X mass spectrometer (Thermo Fisher) operating in data-dependent acquisition (DDA) mode. The full-scan MS spectra were acquired at a resolution of 60000 (at 200 m/z) and ranged from 350 to 1500 m/z with automatic gain control (AGC) target value of 3 × 10^6^ with a maximum ion injection fill time of 20 ms. The 40 most abundant precursor ions were sequentially isolated and fragmented by high-energy collision dissociation (HCD) at a resolution of 15000 (at 200 m/z) with an AGC target value of 5E4, normalized collision energy of 27%, a maximum ion injection time of 80 ms, a dynamic exclusion parameter of 30 s, and an intensity threshold of 1.3E4. The data obtained were combined as the raw data. The search engine Proteome Discoverer 2.2 (PD 2.2, Thermo) was used to analyze the raw MS spectra acquired from the protein database of *D. radiodurans* (strain R1) in UniProt (https://www.UniProt.org/).

### 2.3 Homology Modeling Assay

For homology modeling, the Swiss model was used to predict the structure of DrHU-DNA using the *S. aureus* HU-DNA structure (PDB ID: 4QJU) as the starting model.

### 2.4 Protein Expression and Purification

Wild-type T37A and T37E DrHU were cloned into the modified pET28a expression vector pET28a-HMT, which contains an N-terminal cleavable 6×His tag, as described previously [37]. Transformed E. coli BL21 (DE3) clones were grown in LB broth containing kanamycin at 37°C to an optical density at 600 nm of 0.6-0.8. Protein expression was induced at 37°C by adding isopropyl-β-D-1-thiogalacto- pyranoside (IPTG) at a final concentration of 0.2 mM for 3 h. After harvesting and washing with PBS buffer, the cell pellets were resuspended in buffer A (1 M NaCl, 30 mM Tris-HCl pH 8.0, 5% glycerol, 10 mM imidazole), lysed by sonication, and then centrifuged at 13500 rpm for 35 min at 4°C. The cell debris was discarded, and the supernatant was purified using an AKTA Purifier system. The supernatant was loaded onto a HisTrap HP column (GE Healthcare Biosciences) and eluted with buffer A containing 300 mM imidazole. After tag removal by TEV cleavage, the sample was loaded onto a Ni-NTA column to remove uncleaved protein. After desalting (400 mM NaCl, 30 mM Tris-HCl pH 8.0, 5% glycerol), the flow-through fractions were subjected to a Heparin HP column (GE Healthcare Biosciences) by gradient elution from 400 mM to 2 M NaCl. Proteins were then purified by gel filtration chromatography using a Superdex 75 column (GE Healthcare Biosciences) and eluted with 500 mM KCl, 30 mM Tris-HCl pH 8.0, 5% glycerol, and 1 mM DTT. Purified proteins were concentrated to ∼1 mg/ml for subsequent use and aliquoted in gel filtration buffer, flash frozen and stored at -80°C.

### 2.5 Electrophoretic Mobility Shift Assay

All oligonucleotides labeled with 5’-FAM were purchased from Sangon (Shanghai, China), as listed in Table **S3**. Annealing reactions of duplex DNA were carried out by heating at 95°C for 5 min, followed by slow cooling to room temperature in oligo annealing buffer (30 mM Tris-HCl pH 8.0, 50 mM NaCl, 1 mM DTT). The final reaction mixture consisted of 50 mM NaCl, 20 mM Tris-HCl 8.0, 0.1 mg/ml albumin from bovine serum (BSA), 3 mM DTT, 0.1 mM EDTA, 10% glycerol, 10 mM MgCl_2_, 50 nM duplex DNA and DrHU (0-3200 nM, depending on the *Kd* value). The mixtures were incubated at room temperature for 20 min and separated using 22% native polyacrylamide gels in 1 × TB buffer. The gels were imaged in fluorescence mode (FAM) using a Typhoon FLA 9500 (GE Healthcare Biosciences). Quantifications and curve fitting were performed as described [29]. All reactions were independently repeated at least three times.

### 2.6 Growth Curves and Phenotypic Analyses

Phenotypic assays were performed as previously described [36]. For growth curves, 1 mL cell culture (OD_600_ = 1.0) was resuspended in 50 mL new fresh TGY medium and incubated at 30°C. Cell density was monitored at various incubation times by measuring the OD_600_. For UV treatment, cells at an OD_600_ of 1.0 were diluted and plated on TGY agar plates and illuminated with 100, 200, 300, and 400 J/m^2^ UV radiation. For H_2_O_2_ treatment, cells at an OD_600_ of 1.0 were incubated with 20, 40, 60, and 80 mM H_2_O_2_, diluted and plated on TGY agar plates. Colonies were counted after incubation for 2-3 days. Three independent experiments were performed. Growth and survival fraction curves were plotted using GraphPad Prism 8.

### 2.7 DNA Protection Assay

Wild-type and mutant DrHU proteins (5, 15, 45 µM) were incubated with 1 kb double-stranded linear DNA fragments PCR-amplified from genomic DNA (100 ng) in 20 µl reaction containing 30 mM Tris-HCI pH 8.0 and 100 mM KCl for 30 minutes at room temperature. Next, 150 µM ferrous sulfate and 10 mM hydrogen peroxide were added for 5 minutes at room temperature. The reaction was stopped with buffer containing 0.8% (w/v) SDS and 2.5% glycerol, and the samples were loaded onto a 1% agarose gel in glue (0.5 × TBE). The gel was stained with ethidium bromide and viewed under ultraviolet light.

### 2.8 Statistical Analysis

Results of growth curves, phenotypical analyses, and DNA binding assays were determined using GraphPad Prism 8. The results represented the means and standard deviations (SD) of three independent experiments. One-way ANOVA method followed by Tukey’s post-hoc test was performed to compare the significant differences between wild-type strain and complementary strains.

## 3 RESULTS

### 3.1 Phosphorylation of DrHU at Thr37

**Figure 2:**
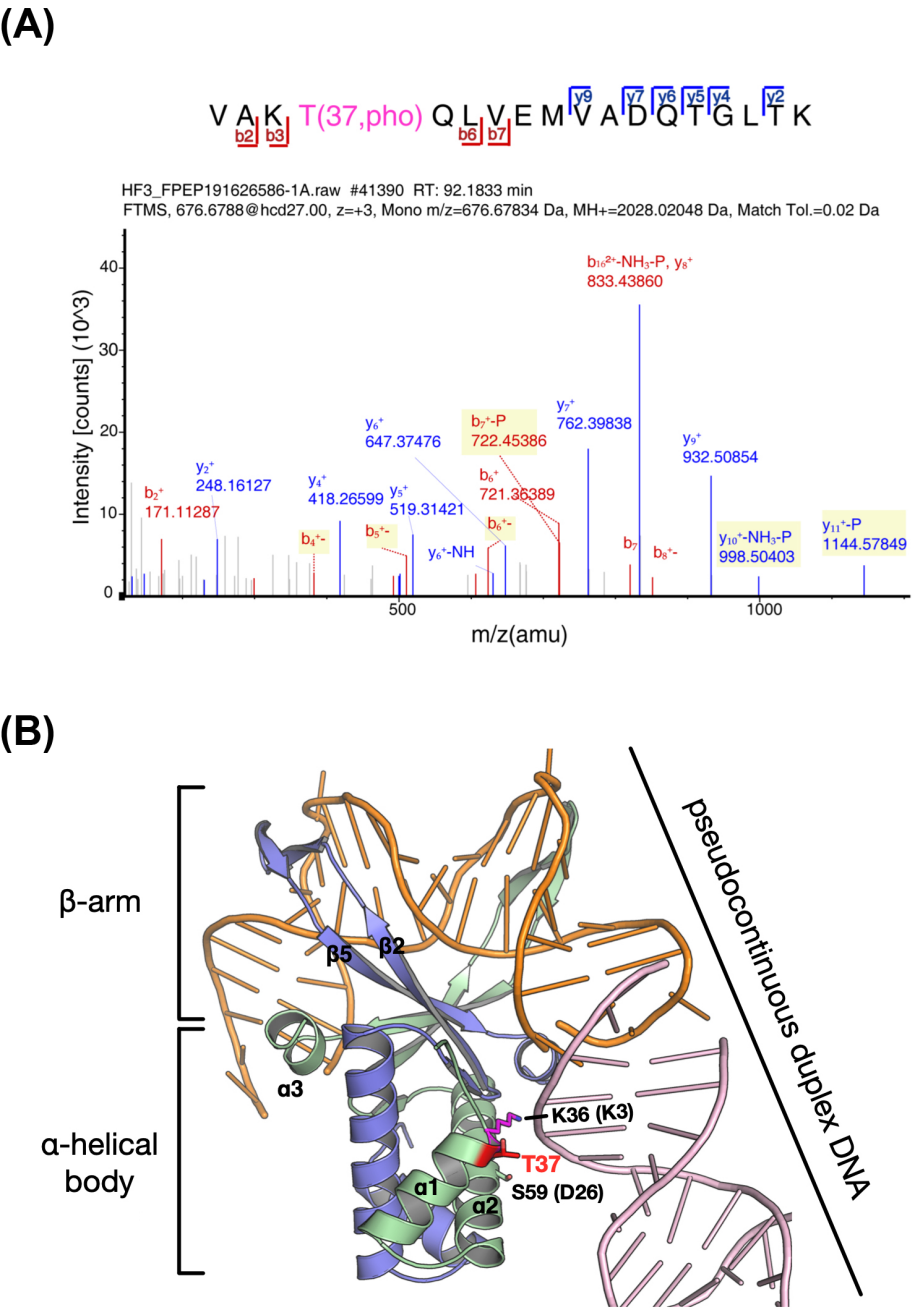
Identification of DrHU phosphorylation. (**A**) LC-MS/MS spectrum of the phosphorylated peptide of DrHU annotated with a comprehensive series of y and b fragment ions. (**B**) The structure of DrHU-DNA was predicted with homology modeling (Swiss model) using the *S. aureus* HU-DNA structure (PDB ID: 4QJU). The DrHU dimer is shown with two protomers and duplex DNA colored green, blue, and orange, respectively. DNA of a neighboring complex forming pseudo-continuous duplex DNA is colored pink. The surface-exposed residues Lys36, Thr37, and Ser59 are shown as sticks, and equivalent residues in *S. aureus* HU are shown in parentheses.

As noted by a previous study, DrHU, containing 122 amino acids, is translated from a natural leaderless mRNA (Figure **1**)). In addition to the HU core domain comprising the DNA-binding residues, the N-terminal lysine-rich extension (residues 1-31) appears to be highly conserved among *Deinococcus* species. Phosphoproteome profiling of *D. radioduran*s under normal conditions was performed to identify possible phosphorylation sites of DrHU. After enrichment of phosphopeptide, LC–MS/MS (liquid chromatography-tandem mass spectrometry) analysis showed that DrHU contained a phosphorylation modification at the Thr37 site, which has a mass shift of +79.9663 Da based on a search against the UniProt database (Figures **2A** and **S1**). This phosphorylation site is located at the N-terminal region of the core domain, which is present in not only all *Deinococcus* species but also HU proteins from other bacteria, *e.g.*, threonine in HU from *Staphylococcus aureus* and threonine/serine in *E. coli* HUα/β (Figure **1B**). Notably, a lysine residue (Lys36 in DrHU and Lys3 in SauHU) upstream of Thr37 is conserved in almost all HU proteins, which undergoes acetylation modification in *M. tuberculosis* HU protein (Figures **1B** and **S2**).

### 3.2 The DrHU Mutant Mimicking Phosphorylation Shows Enhanced Duplex DNA Binding

A homology model of the DrHU-DNA complex was predicted using the *S. aureus* HU-DNA structure (PDB ID: 4QJU) (Figure **2B**). Despite being biologically irrelevant, the phosphorylation site Thr37 of DrHU is exposed on the surface and interacts with the DNA of a symmetric-related HU-DNA complex. Lys36, the conserved residue adjacent to the phosphorylation site, interacts with Ser59 (equivalent to Asp26 of *S. aureus* HU). It has been proposed that this Lys-Asp salt bridge interaction of *S. aureus* HU is maintained upon DNA binding [17]. To explore whether phosphorylation has an effect on its function, we expressed and purified DrHU and checked its DNA-binding properties using different lengths of duplex DNA (Table **1** and Figure **S3**). In addition to the wild-type protein, we generated two types of mutant proteins: one in which this threonine is replaced by glutamate to mimic a permanent state of phosphorylation (T37E), and one in which this threonine is mutated to alanine to prevent phosphorylation (T37A).

Although the binding affinity was rather low, wild-type DrHU was able to form a stable single complex with duplex DNA in a length-dependent manner. Incubation of DrHU with a short DNA duplex (24 bp and 36 bp) showed a dissociation constant (*Kd*) in the millimolar range (Table **1** and Figure **S4**). When longer duplex DNA was tested, DrHU exhibited relatively higher binding affinity (449.3 ± 29.6 nM for 48-bp duplex DNA). However, no complex was detectable with the phosphorylation-defective T37A mutant protein incubated with 24-, 36- or 48-bp duplex DNA, indicating that Thr37, *per se*, is required for the DNA-binding activity of DrHU. Compared to the wild-type protein, the phosphorylation mimic DrHU (T37E) showed increased DNA-binding abilities with all duplex DNA tested, especially for longer duplex DNA, which was approximately 2.9-fold higher than that for 36-bp duplex DNA (457.4 ± 62.9 nM for T37E *vs*. 1352.0 ± 200.5 nM for wild-type).

### 3.3 Phenotypical Analysis of the Phosphorylation-mimicking DrHU Mutant

**Figure 3:**
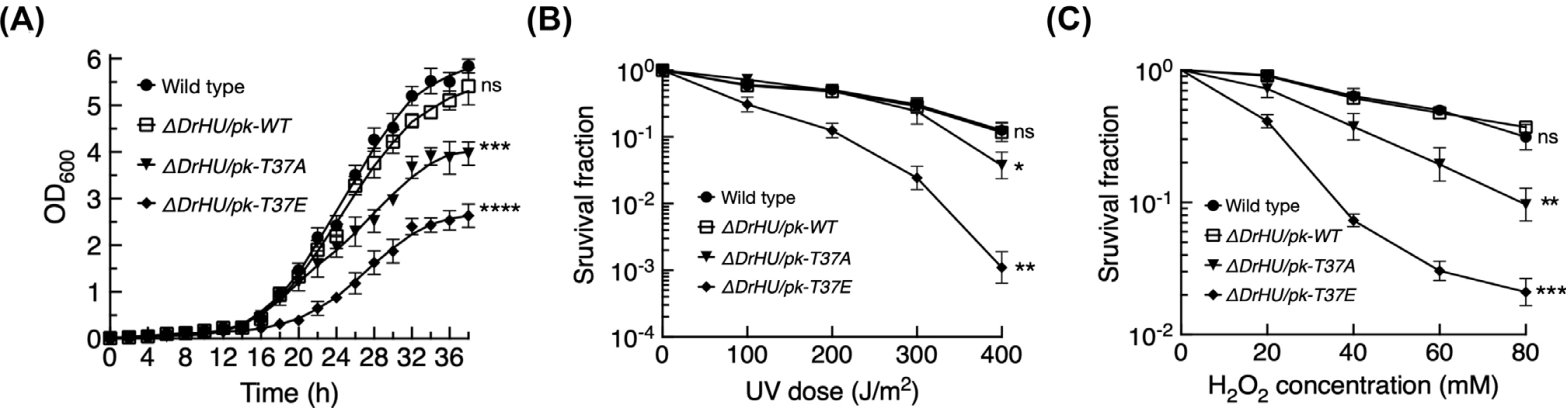
Phenotypic analyses of DrHU complementation-knockout strains. (**A**) Growth curves of wild-type (filled circles), *ΔDrHU/pk-WT* (open squares), *ΔDrHU/pk-T37A* (filled triangles), and *ΔDrHU/pk-T37E* (filled squares). (**B** and **C**) Phenotypic analyses of wild-type (filled circles), *ΔDrHU/pk-WT* (open squares), *ΔDrHU/pk-T37A* (filled triangles), and *ΔDrHU/pk-T37E* (filled squares) following UV radiation (100-400 J/m^2^) and H_2_O_2_ (20-80 mM) treatments. One-way ANOVA method followed by Tukey’s post-hoc test was performed to compare the significant differences between the wild-type strain and complementary strains. All the data represent the means of the three replicates (bars represent standard deviations; ns: not significant, *p < 0.05, **p < 0.01, ***p < 0.001, ****p < 0.0001).

As noted by previous studies, complete inactivation of DrHU is lethal [28]. We were unable to generate a homozygous knockout DrHU mutant using a conventional homologous recombination-based method. To investigate the phenotypical properties of DrHU phosphorylation with regards to DNA repair, we used a complemented-knockout strategy instead of a knockout-complemented method. Briefly, the complementary plasmid pRADK containing *DR_A0065* (encoding wild-type (WT), T37A, and T37E DrHU) was transformed into the *D. radioduran*s wild-type R1 strain, followed by knockout mutagenesis as described above. Compared to the wild-type R1 strain, the WT complemented-knockout strain (*ΔDrHU/pk-WT*) did not show growth defects (Figure **3A**) and displayed no sensitivity to DNA-damaging agents, including ultraviolet (UV) irradiation (Figure **3B**) and hydrogen peroxide (H_2_O_2_) (Figure **3C**), indicating that genomic-expressed DrHU can be fully complemented by plasmid-based copies. In addition to reduced growth (Figure **3A**), the T37A (*ΔDrHU/pk-T37A*) and T37E (*ΔDrHU/pk-T37E*) complemented-knockout strains were both sensitive to DNA-damaging agents to various degrees (Figures **3B** and **C**). Interestingly, although the survival rates of *ΔDrHU/pk-T37A* were 1-2 orders of magnitude lower than those of the wild-type strain in the presence of high DNA-damaging agent concentrations, the phosphorylation mimic *ΔDrHU/pk-T37E* displayed survival rates that were more than three orders of magnitude lower (Figures **3B** and **C**). Given the enhanced duplex DNA-binding capability of T37E DrHU, these results suggest the toxic effects of over phosphorylation of DrHU *in vivo.*

### 3.4 DNA Protection by DrHU

**Figure 4:**
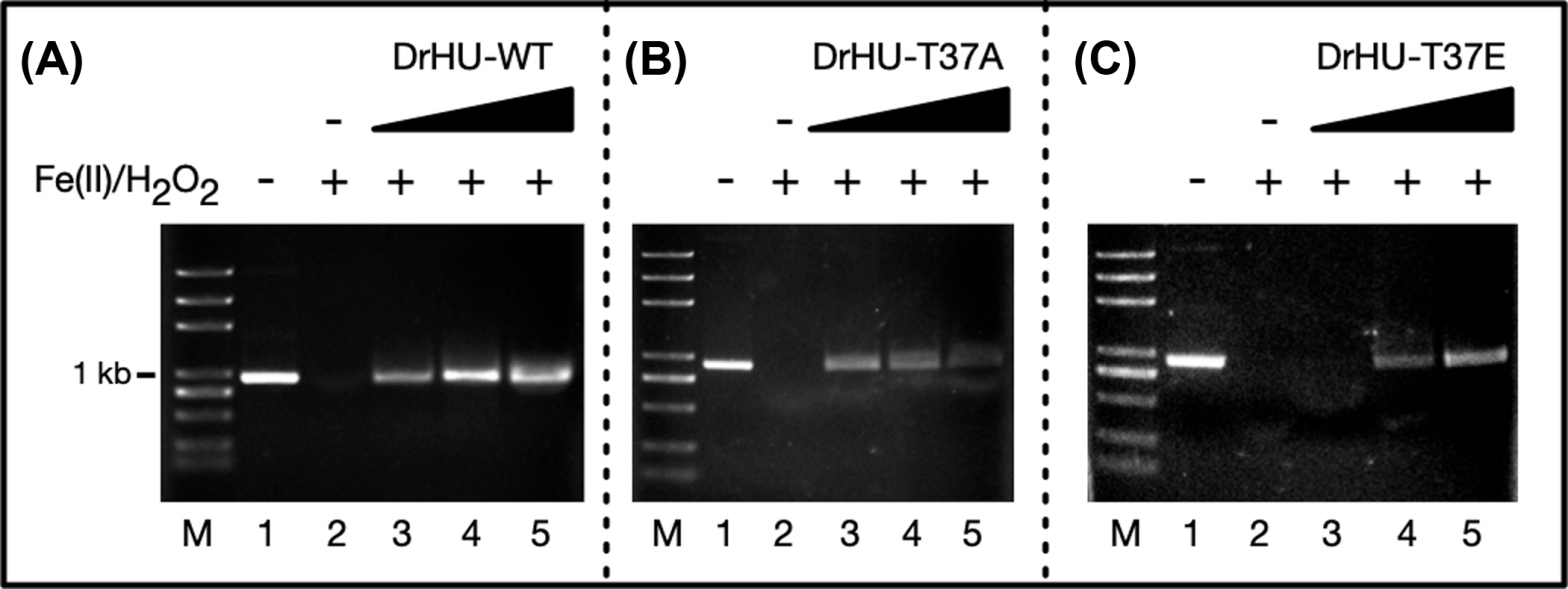
Protection of DNA by DrHU. Cleavage of 100 ng of 1-kb duplex DNA by the Fenton reaction (Fe(NH_4_)_2_(SO_4_)_2_ and H_2_O_2_) is denoted above the panels, along with the absence of DrHU (Lane 2) or the presence of increasing concentrations (Lane 3: 5 µM; Lane 4: 15 µM; Lane 5: 45 µM) of wild-type (**A**), T37A (**B**), and T37E (**C**) DrHU proteins, respectively.

As HU proteins have been reported to protect DNA against hydroxyl radical attack, we next examined whether phosphorylation of DrHU affects its DNA protection features. A 1-kb linear DNA duplex was incubated with Fe(NH_4_)_2_(SO_4_)_2_ and H_2_O_2_ (hydroxyl radical produced by the Fenton reaction) in the presence or absence of DrHU (Figure **4**)). Linear DNA was completely degraded in the absence of DrHU (Figure **4A**, Lane 2), but it was substantially protected by subsequent addition of wild-type DrHU (Figure **4A**, Lanes 3-5). A similar protective effect was also observed with the T37A mutant protein at low concentration (Figure **4B**). However, the phosphorylation mimic T37E exhibited much weaker protection against hydroxyl radical cleavage, and the linear DNA was entirely degraded at a low concentration of T37E mutant protein (Figure **4C**, Lanes 3).

## 4 DISCUSSION

Architectural changes in chromosomes appear to be one of the most universal and effective strategies for the environmental adaptation of bacteria. Similar to eukaryotic organisms, bacterial chromosomes are highly condensed, and form compartment-like structures called nucleoids. Bacteria express a large family of nucleoid-associated proteins (NAPs) orchestrating nucleoid dynamics. HU proteins, the most abundant NAP in some bacteria, contribute to both nucleoid organization and gene expression under certain conditions, such as environmental stress and virulence [38, 39]. *D. radioduran*s can withstand extreme conditions of irradiation, desiccation, and oxidative stress. Despite the debate regarding the ring-shaped aspect of *D. radioduran*s nucleoids, it is well accepted that keeping nucleoids condensed and preventing unrepaired chromosome segregation under conditions of stress are critical for the robustness of this bacterium [40]. Such condensed nucleoids might passively facilitate DNA repair processes. Unlike redundant HU proteins in other *Deinococcus* species, *e.g.*, *Deinococcus deserti*, DrHU appears to be the only HU protein present in *D. radioduran*s, and is required for the maintenance of condensed nucleoid architecture. However, the regulatory mechanisms of DrHU remain unclear.

In addition to gene expression, PTM plays key roles in the regulation of protein structure, biochemical activity, and protein-protein interactions. Phosphorylation, one of the most important and well-studied PTMs, is involved in nucleoid/mitochondrial nucleoid organization in both bacterial and eukaryotic organisms. In this study, the sole phosphorylation site Thr37 of DrHU was identified; it is adjacent to a lysine residue (Lys3 in other bacteria) highly conserved among HU proteins (Figure **1B**). Interestingly, this lysine is not only required for effective short-length DNA wrapping of HU but is also involved in the regulation of DNA compaction through acetylation in *M. tuberculosis* HU [16]. Regardless of the small number of studies on the crosstalk between different PTMs, serine/threonine phosphorylation has an effect on neighboring lysine acetylation as well as its function. In addition to the enhanced electrostatic interaction between the amino group of the lysine side chain and the negatively charged phosphate group of modified serine/threonine, phosphorylation might also alter the lysine acetylation level both *in vivo* and *in vitro*. For example, phosphorylation of Ser10 at histone H3 enhances acetylation at Lys14, contributing to gene regulation [41]. Moreover, such an augmenting effect might occur even at a considerable distance [42]. Although it remains unclear whether DrHU is acetylated, its phosphorylation may also modulate its acetylation modifications, leading to altered DNA-binding capability.

According to the original annotation, DrHU contains 137 amino acids. Given the suggested leaderless translation *in vivo*, DrHU was later reannotated to be 15 residues shorter than initially reported. We were able to express and purify both wild-type and phosphorylation mimic DrHU (protein length of 122 amino acids) to assess their DNA-binding capabilities (Table **1**). Wild-type DrHU was able to form a stable complex with a binding site of 24 bp of short duplex DNA, which is generally comparable with HU homologous from other bacteria, *e.g.*, 37 bp for *E. coli* TF1 and 19 bp for *Helicobacter pylori* HU [21, 43]. However, the binding affinity (mM range) for short DNA duplexes was extremely low, and significantly enhanced DNA-binding capabilities were observed with increased DNA length. This might be explained by interactions between DrHU and pseudo-continuous duplex DNA (Figure **2B**, modeled using *S. aureus* HU-DNA structure 4QJU), which is consistent with the proposed DNA-binding role of the α-helical body of HU proteins [44]. Moreover, DNA binding of T37A mutant DrHU was almost eliminated even with a longer DNA (48-bp duplex DNA). These results suggest that the surface-exposed threonine at this position directly contributes to the DNA binding of DrHU. In contrast, T37E DrHU exhibited much elevated DNA binding for longer duplex DNA. It is accepted that a large amount of salt bridge formation and disruption usually accompanies DNA binding by HU proteins [17, 44]. In the case of *S. aureus* HU, an internal salt bridge between Lys3 (Lys36 in DrHU) and Asp26 was observed [17]. However, the equivalent Asp residue is Ser in *Deinococcus* HU proteins (Ser59 in DrHU). Thus, the enhanced DNA-binding capability of T37E may be due to a possible salt bridge formation between Lys36 and phosphorylated Thr37, modulating the DNA-binding process of DrHU.

In addition to DNA compaction, HU proteins can form DNA-protein-DNA bridges and protect DNA from digestion, regulating DNA transcriptions and facilitating DNA repair. For example, HU proteins from *E. coli* and *Bacillus subtilis* favor DNA recombination and repair intermediates. *D. radioduran*s, the model organism for DNA repair studies, has the extraordinary capability to repair hundreds of DNA double-strand breaks (DSBs) after heavy irradiation. Compared to other types of DNA damage, DSBs are considered the most lethal form, lacking an intact undamaged template strand for repair. Thus, preventing diffusion of these unrepaired DSBs appears to be a promising strategy for *D. radioduran*s to survive under extreme conditions. Given that depletion of DrHU causes diffuse nucleoid structure, we performed phenotypical assays to evaluate the possible effect of DrHU phosphorylation in the absence of a gene encoding DrHU. Consistent with the almost abolished short DNA-binding capability, *ΔDrHU/pk-T37A* exhibited growth defects and was sensitive to environmental stresses (Figure **3**)). Unexpectedly, the phenotypes of *ΔDrHU/pk-T37E* were much more severe than those of *ΔDrHU/pk-T37A*. These toxic effects might be due to the following: (1) in the absence of wild-type DrHU, although the enhanced DNA binding of T37E DrHU may facilitate restraining DSBs after DNA damage, this phosphorylation-mimic protein is defective in normal dissociation from DNA, leading to suppression of genes involved in DNA repair and chromosome segregation; (2) compared to wild-type DrHU, T37E shows much reduced protective effects against oxidative attack, which can easily cause DNA damage; and (3) the HU protein has been shown to regulate the formation of higher-order protein complexes involved in DNA replication and gene expression by interactions with multiple protein partners [45]. Phosphoryla- tion may also alter the protein-protein interactions of DrHU.

## CONCLUSION

Collectively, our findings provide the first evidence that phosphorylation modulates the DNA-binding capabilities of the histone-like HU protein from *D. radioduran*s, which may contribute to the robustness of this organism. However, physiological phosphorylation is dynamic and transient in response to various stimuli or cellular events. Given the toxicity of the phosphorylation-mimic protein in the absence of genomic-expressed wild-type DrHU, phosphorylation of DrHU must be very tightly controlled in response to environmental stresses.

## Figures and Tables

**Table 1:** Dissociation constant *Kd* (nM) of DrHU with duplex DNA of various lengths. All reactions were independently repeated three times.

**DNA Length**	**Protein**	***Kd* (nM)**
24-bp	WT	1478.0 ± 41.8
	T37E	1180.0 ± 95.9
36-bp	WT	1352.0 ± 200.5
	T37E	457.4 ± 62.9
48-bp	WT	449.3 ± 29.6
	T37E	388.6 ± 56.0

## Data Availability

Not applicable.

## LIST OF ABBREVIATIONS

PTMs: Posttranslational Modifications
TEAB: Triethylammonium Bicarbonate
DDA: Data-dependent Acquisition
HCD: High-energy Collision Dissociation
SD: Standard Deviations
WT: Wild-type
NAPs: Nucleoid-associated Proteins
