# Supplementary material for: Phosphorylation Regulation of a Histone-like HU Protein from Deinococcus radiodurans
Source: Protein Pept Lett. 2022 Nov 8;29(10):891–9. doi: 10.2174/0929866529666220819121911 (PMC9900698; doi:10.2174/0929866529666220819121911)

Supplementary Material

Phosphorylation Regulation of a Histone-like HU Protein from *Deinococcus radiodurans*

Jinfeng Hou[1], Jingli Dai[1], Zijing Chen[1], Yudong Wang[1], Jiajia Cao[1], Jing Hu[1], Shumai Ye[1] and Yuejin Hua[1] and Ye Zhao[1,*]

[1]*MOE Key Laboratory of Biosystems Homeostasis & Protection, College of Life Sciences, Zhejiang University, Hangzhou 310000, China*

Table S1. strains and plasmids used in this study

Strain and plasmid	Description	Source
**Strains**		
E. coli		
DH5α	*E. coli* cloning strain	TransGene
BL21 (DE3)	*E. coli* expression strain	TransGene
D. radiodurans		
R1	wild-type strain	ATCC13939
ΔDrHU/pk-T37A	*DR_A0065* compensated with pRAD-DrHU(T37A)	This study
ΔDrHU/pk-T37E	*DR_A0065* compensated with pRAD-DrHU(T37E)	This study
ΔDrHU/pk-WT	*DR_A0065* compensated with pRAD-DrHU(WT)	This study
**Plasmids**		
pRADK	*E. coli–D. radiodurans* shuttle vector	Dai et al., 2020
pRAD-DrHU(WT)	pRADK but kan^r was replaced with *DR_A0065*	This study
pRAD-DrHU(T37E)	pRADK but kan^r was replaced with *DR_A0065* (T37E)	This study
pRAD-DrHU(T37A)	pRADK but kan^r was replaced with *DR_A0065* (T37A)	This study
pET28a-HMT(WT)	pET28 plasmid modified with a maltose binding protein and a TEV protease site, ligated with *DR_A0065*	This study
pET28a-HMT(T37E)	pET28 plasmid modified with a maltose binding protein and a TEV protease site, ligated with *DR_A0065* (T37E)	This study
pET28a-HMT(T37A)	pET28 plasmid modified with a maltose binding protein and a TEV protease site, ligated with *DR_A0065* (T37A)	This study

Table S2. Primers used in this study

**Primers**	**Sequences (5′-3′)**
pRAD-*DR_A0065*-F (NdeI)	CTCACAGGAGGACCCCATATGACGAAAAAGTCTACCAAGGCC
pRAD-*DR_A0065*-R (BamHI)	CCTGCAGGTCGAATCGGATCCTTACAGGTTGCCCTTGAGGG
pRAD-*DR_A0065*-T37A-F	GCAAGGTCGCCAAGGCACAGCTGGTCGAAATG
pRAD-*DR_A0065*-T37A-R	CATTTCGACCAGCTGCACCTTGGCGACCTTGC
pRAD-*DR_A0065*-T37E-F	GCAAGGTCGCCAAGGAACAGCTGGTCGAAATG
pRAD-*DR_A0065*-T37E-R	CATTTCGACCAGCTGTTCCTTGGCGACCTTGC
DR_A0065 (upstream)-F	CGCCCTGGTTGTCGAAGGT
DR_A0065 (upstream)-R (BamH I)	CGGGATCCGGTGAGCAGCATGACATATCTCTT
DR_A0065 (downstream)-F (Hind III)	CCAAGCTTGCGGGTTCGTCCCTGAC
DR_A0065 (downstream)-R	GCTGCCCACCACGATGAC
str-F (BamH I)	CGGGATCCAAGCTTGATATCGAATTCGAGCTC
str-R (Hind III)	CCAAGCTTGGATCCTTATTTGCCGACTACCTT
pET28a-*DR_A0065*-F	CCAGGAGGCAGCCATCATTGGATGACGAAAAAGTCTACCAAGGC
pET28a-*DR_A0065*-R (BamHI)	ACGGAGCTCGAATTCGGATCCTTACAGGTTGCCCTTGAGGGTGCT
pET28a-*DR_A0065*-T37A-F	GCAAGGTCGCCAAGGCACAGCTGGTCGAAATG
pET28a-*DR_A0065*-T37A-R	CATTTCGACCAGCTGATCCTTGGCGACCTTGC
pET28a-*DR_A0065*-T37E-F	GCAAGGTCGCCAAGGAACAGCTGGTCGAAATG
pET28a-*DR_A0065*-T37E-R	CATTTCGACCAGCTGTTCCTTGGCGACCTTGC

Table S3. Oligonucleotide sequences

Name	Sequences (5′-3′)
Duplex DNA (24bp)	TCAATTATGTCAATGACATAAAAG<br>CTTTTATGTCATTGACATAATTGA
Duplex DNA (36bp)	GCAGAGTCAATTATGTCAATGACATAAAAGGCTCAG<br>CTGAGCCTTTTATGTCATTGACATAATTGACTCTGC
Duplex DNA (48bp)	TAACAAGCAGAGTCAATTATGTCAATGACATAAAAGGCTCAGGCCTTC<br>GAAGGCCTGAGCCTTTTATGTCATTGACATAATTGACTCTGCTTGTTA
1-kb duplex DNA	AAGCTTGCCACCATGTACCCATACGACGTACCAGATTACGCTTTCATCA<br>ACGGCTACGCCCCTCAGAATCTGAGCATCAGAGGCGAGTACCAGATCAA<br>CTTTCACATCGTGAACTGGAATCTGTCCAACCCTGACCCCACCTCCAGC<br>GAGTACATCACCCTGCTGAGAGATATCCAGGACAAGGTGACAACACTGT<br>ACAAGGGCTCCCAGCTGCACGATACCTTCAGATTTTGCCTGGTGACAAA<br>TCTGACAATGGATTCCGTGCTGGTGACCGTGAAGGCCCTGTTTAGCTCC<br>AACCTGGACCCCTCCCTGGTGGAGCAGGTGTTCCTGGACAAGACACTGA<br>ACGCCAGCTTTCACTGGCTGGGCTCCACCTACCAGCTGGTGGACATCCA<br>CGTGACAGAGATGGAGAGCAGCGTGTACCAGCCTACCAGCTCCTCCAGC<br>ACCCAGCACTTTTACCTGAATTTCACAATCACCAACCTGCCTTACAGCC<br>AGGACAAGGCCCAGCCCGGCACAACCAACTACCAGAGAAACAAGAGGAA<br>TATCGAGGACGCCCTGAATCAGCTGTTCAGGAACTCCAGCATCAAGTCC<br>TACTTTTCCGATTGCCAGGTGTCCACCTTTAGAAGCGTGCCTAATAGAC<br>ACCACACCGGCGTGGATAGCCTGTGTAATTTTTCCCCCCTGGCCAGAAG<br>AGTGGACAGGGTGGCCATCTACGAGGAGTTTCTGAGAATGACCAGAAAC<br>GGCACCCAGCTGCAGAATTTTACCCTGGATAGAAGCTCCGTGCTGGTCG<br>ATGGCTACAGCCCTAACAGAAATGAGCCCCTGACCGGCAATTCCGATCT<br>GCCTTTCTGGGCCGTGATCCTGATCGGCCTGGCCGGCCTGCTGGGCGTT<br>ATCACATGCCTGATCTGTGGCGTGCTGGTGACAACAAGAAGGAGGAAGA<br>AGGAGGGCGAGTACAATGTGCAGCAGCAGTGCCCCGGCTACTACCAGTC<br>CCACCTGGACCTGGAGGATCTGCAGGATTACAAGGACGACGATGACAAG

Figure S1. Identification of DrHU phosphorylation that contains the raw data.

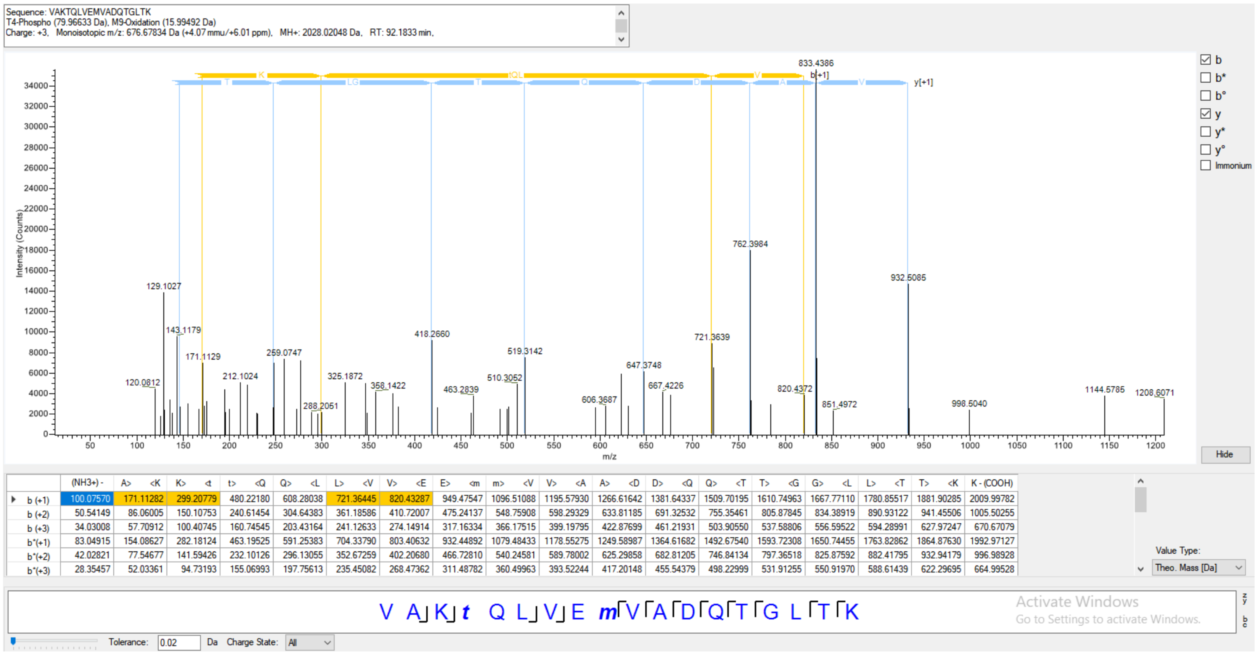

	(NH3+) -	A> <K	K> <t	t> <Q	Q> <L	L> <V	V> <E	E> <m	m> <V	V> <A	A> <D	D> <Q	Q> <T	T> <G	G> <L	L> <T	T> <K	K - (COOH)
b (+1)	100.07570	171.11282	299.20779	480.22180	608.28038	721.36445	820.43287	949.47547	1096.51088	1195.57930	1266.61642	1381.64337	1509.70195	1610.74963	1667.77110	1780.85517	1881.90285	2009.99782
b (+2)	50.54149	86.06005	150.10753	240.61454	304.64383	361.18586	410.72007	475.24137	548.75908	598.29329	633.81185	691.32532	755.35461	805.87845	834.38919	890.93122	941.45506	1005.50255
b (+3)	34.03008	57.70912	100.40745	160.74545	203.43164	241.12633	274.14914	317.16334	366.17515	399.19795	422.87699	461.21931	503.90550	537.58806	556.59522	594.28991	627.97247	670.67079
b*(+1)	83.04915	154.08627	282.18124	463.19525	591.25383	704.33790	803.40632	932.44892	1079.48433	1178.55275	1249.58987	1364.61682	1492.67540	1593.72308	1650.74455	1763.82862	1864.87630	1992.97127
b*(+2)	42.02821	77.54677	141.59426	232.10126	296.13055	352.67259	402.20680	466.72810	540.24581	589.78002	625.29858	682.81205	746.84134	797.36518	825.87592	882.41795	932.94179	996.98928
b*(+3)	28.35457	52.03361	94.73193	155.06993	197.75613	235.45082	268.47362	311.48782	360.49963	393.52244	417.20148	455.54379	498.22999	531.91255	550.91970	588.61439	622.29695	664.99528

Figure S2. Alignment of HU proteins from different organisms including *Deinococcus* species (*Deinococcus radiodurans*, *Deinococcus wulumiqiensis*, and *Deinococcus geothermalis*), *Mycobacterium tuberculosis*, *Staphylococcus aureus*, and *Escherichia coli*.

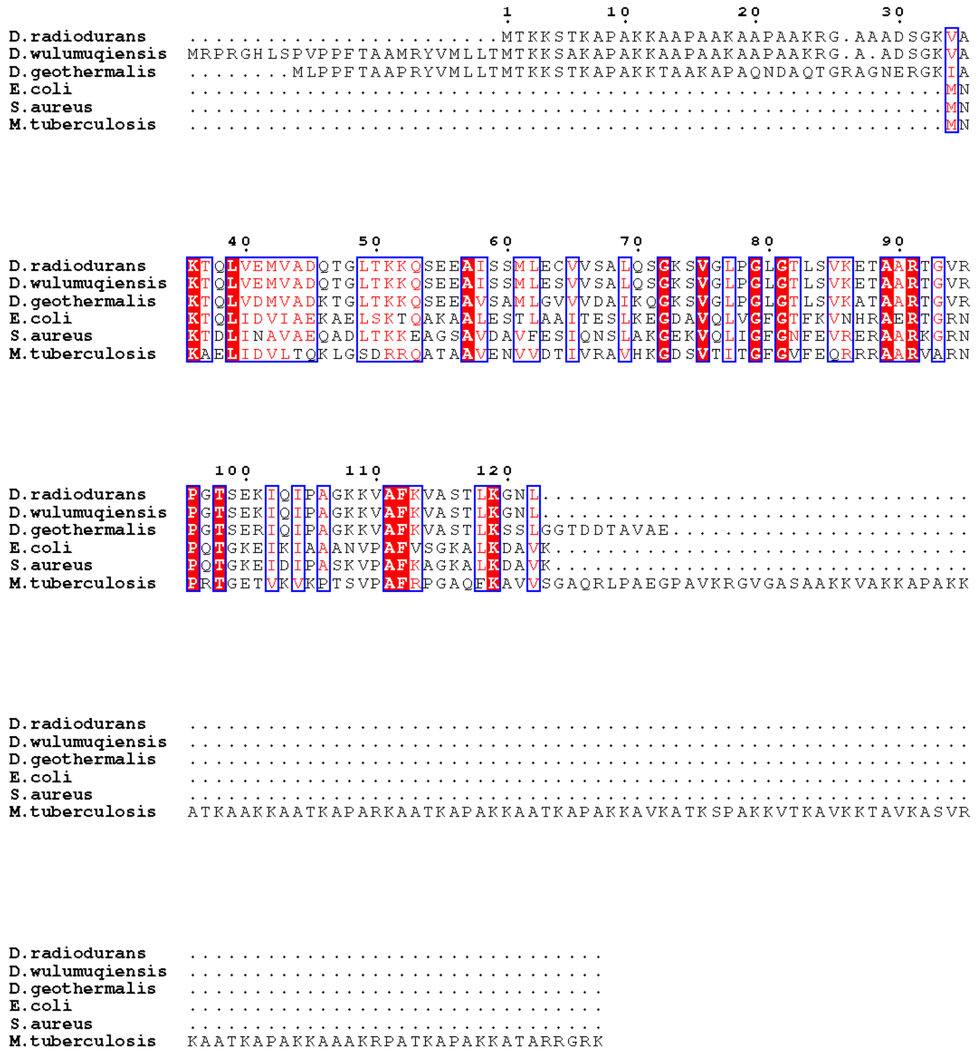

Figure S3. A gel showing purified wild-type DrHU protein.

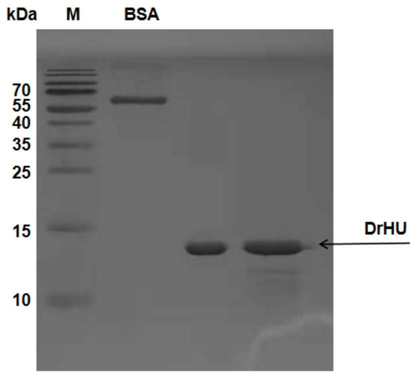

Figure S4. Gels showing EMSA results of WT, T37E, and T37A DrHU incubated with 24-, 36- or 48-bp duplex DNA. M is the marker. The DNA:protein ratios are shown on top.

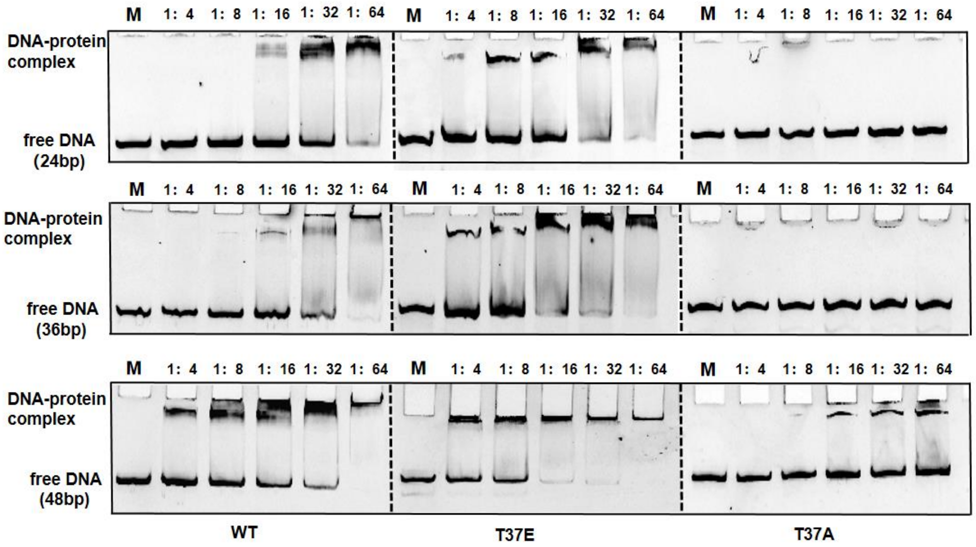

Supplement: Supplementary file 1 [file PPL-29-891_SD1.pdf]
